# 2316. Impact of Surveillance and Decolonization on Methicillin-resistant Staphylococcus aureus Infection Rates in a Neonatal Intensive Care Unit

**DOI:** 10.1093/ofid/ofac492.148

**Published:** 2022-12-15

**Authors:** Nahid Hiermandi, Catherine Foster, Krystal Purnell, Judith R Campbell, Lucila Marquez

**Affiliations:** Baylor College of Medicine, Houson, TX; Baylor College of Medicine, Houson, TX; Texas Children's Hospital, Houston, Texas; Baylor College of Medicine, Houson, TX; Baylor College of Medicine, Houson, TX

## Abstract

**Background:**

Neonates colonized with methicillin-resistant *Staphylococcus aureus* (MRSA) are at high risk of developing life-threatening MRSA infection. In neonatal intensive care units, surveillance for MRSA colonization and decolonization of positive infants aims to interrupt MRSA transmission and progression to infection.

**Methods:**

In this retrospective study, we compared rates of MRSA infections before and after implementation of an MRSA surveillance and decolonization protocol in a 42-bed neonatal intensive care unit. Infections from Jan 2014 to Dec 2020 were reviewed. The protocol was introduced in late October 2016 and fully implemented by Feb 2017. MRSA colonization was initially assessed with cultures of three body sites and later the methodology was changed to PCR. Colonized infants were treated with topical mupirocin and 2% chlorhexidine gluconate wipes. MRSA infection was defined as any specimen from which MRSA was isolated. Multiple positive specimens from the same site were considered the same infection if within a 14-day window. Infection rates were calculated as unique infections per 1000 patient days. An interrupted time-series analysis was performed on Stata 14.2. This study was approved through the Baylor College of Medicine Institutional Review Board.

**Results:**

While we continued to identify colonized infants in the unit, monthly infection rates in the post-intervention period never reached the peak rates in the pre-intervention period and clusters of infections have been eliminated (Figure 1). Prior to the intervention in Feb 2017, the rate of infection increased by 0.06 infections per 1000 patient days every month (p=0.124, CI = -0.017, 0.137). During the first month after full implementation of the intervention, the infection rate decreased by 1.522 infections per 1000 patient days (p=0.145, CI = -3.579, 0.534) followed by a monthly rate decrease of 0.066 infections per 1000 patient days (p=0.088, CI = -0.142, 0.010) (Figure 2).

**Conclusion:**

We found a sustained trend towards decreased MRSA infections after implementing MRSA surveillance and decolonization. Further studies should explore adherence to decolonization, whether decolonization is enduring and what risk factors lead to recurrence of colonization.

**Disclosures:**

**All Authors**: No reported disclosures.

**Figure 1:**
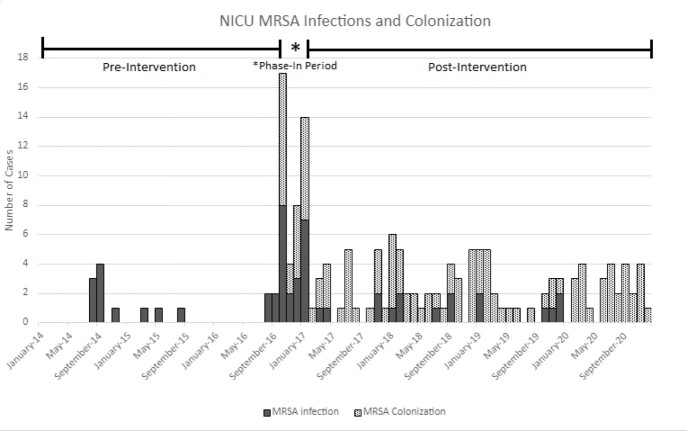
Cases of MRSA infections before and after implementing MRSA surveillance and decolonization protocol

**Figure 2:**
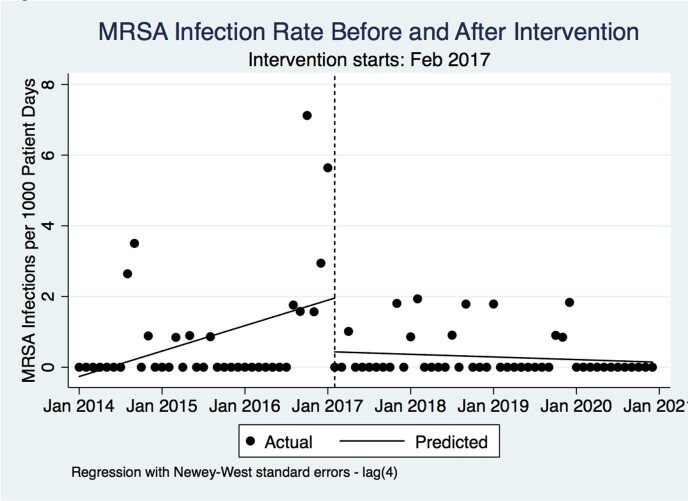
MRSA Infection Rates Before and After Intervention from Interrupted Time Series Analysis

